# Group peer mentoring is effective for different demographic groups of biomedical research faculty: A controlled trial

**DOI:** 10.1371/journal.pone.0300043

**Published:** 2024-03-18

**Authors:** Linda H. Pololi, Arthur T. Evans, Janet T. Civian, Tay McNamara, Robert T. Brennan

**Affiliations:** 1 National Initiative on Gender, Culture and Leadership in Medicine: C-Change, Institute for Economic and Racial Equity, The Heller School for Social Policy and Management, Brandeis University, Waltham, Massachusetts, United States of America; 2 Section of Hospital Medicine, Weill Cornell Medicine, New York, New York, United States of America; Medical College of Wisconsin - Central Wisconsin Campus, UNITED STATES

## Abstract

**Introduction:**

Improved mentoring of midcareer researchers in medical schools has been identified as an important potential avenue for addressing low vitality and high burnout rates in faculty, and the scarcity of both underrepresented minority (URM) faculty and women in biomedical research. To address the need for widescale effective mentoring, we sought to determine whether a group peer mentoring intervention (C-Change Mentoring and Leadership Institute) for early midcareer research faculty was effective for different demographic groups in a controlled trial.

**Methods and materials:**

Thirty-five diverse early midcareer faculty and 70 propensity-matched (PM) control subjects matched to intervention subjects on a) study inclusion criteria; b) gender, race, and ethnicity, degree, rank, years of experience, publications, grants; and c) pretest survey outcome variables, participated in the intervention. The C-Change Participant Survey assessed vitality, self-efficacy in career advancement, research success, mentoring others, valuing diversity, cognitive empathy, and anti-sexism/anti-racism skills at pretest and intervention completion. Analysis using multiple regression models included outcome pretest values and indicator variables for intervention, gender, URM status, and MD vs. PhD. Hypotheses regarding differential effectiveness of the intervention by demographic group were tested by including cross-product terms between the demographic indicator variables and the intervention indicator. Missing data were addressed using chained equations to create 100 data sets.

**Results and discussion:**

The intervention participants had significantly higher (favorable) scores than PM controls for: self-assessed change in vitality; self-efficacy for career advancement, research, and mentoring others; cognitive empathy; and anti-sexism/racism skills. The benefits of the intervention were nearly identical across: gender, URM vs non-URM faculty, and degree MD/PhD, except vitality significantly increased for non-URM subjects, and not for URM faculty. Self-assessed change in vitality increased for URM and non-URM.

**Conclusion:**

The intervention worked successfully for enhancing vitality, self-efficacy and cross-cultural engagement across different demographic groups of biomedical research faculty.

## Introduction

Academic medicine is plagued by faculty burnout, low vitality, and attrition [1–7]. Furthermore, faculty with historically underrepresented racial and ethnic backgrounds [8–12] and female faculty [13–19] face difficulties in professional advancement in medical schools and teaching hospitals, and in obtaining NIH research funding [20]. Mentoring is widely recommended to address these challenges. Even so, few evidence-based mentoring programs are reported in the literature. Formal mentoring efforts by medical schools for their faculty, most of which use one-on-one senior faculty mentoring a junior faculty member, have resulted in a mere 30% of faculty nationally reporting good mentoring [21].

Seeking to address the need for widescale mentoring that is feasible and effective for male and female faculty from underrepresented and non-underrepresented groups, we developed a facilitated group peer mentoring model: the *C-Change Mentoring & Leadership Institute*. Aims of the intervention were to positively impact faculty career advancement, vitality, self-efficacy, and cross-cultural competence: areas considered essential for career advancement and effective leadership in academic medicine. The mentoring model implemented the experience of a novel culture of learning for medical school faculty. We conducted a randomized controlled study (RCT) of the mentoring model which demonstrated its overall efficacy for research faculty [22]. The RCT compared overall outcomes for an intervention faculty group with faculty who did not receive the intervention. Building on that study, we prospectively more than doubled sample size and used identical outcome measures to allow us in this study to analyze whether the mentoring intervention is effective for different demographic groups of early midcareer research faculty in academic medicine. In this study we compare two cohorts of intervention subjects with a significantly larger propensity-matched control group, allowing us to analyze any differential effect of the intervention on groups of faculty by gender, race and ethnicity and degree demographic.

Early midcareer physician scientists and PhD investigators were selected for inclusion in this study since 1) there is marked attrition from NIH funded research after the initial R01 [23] and 2) inclusive science needs a diverse midcareer faculty population. Research faculty often fill leadership roles in academic health centers; having these faculty drawn from different demographic groups would help academic medicine diversify its leadership to more accurately represent the skills, broad experiences, and differing perspectives in those who hold organizational power and decision-making authority.

## Materials and methods

### Recruitment of study participants

In 2019–2020, (from October 16, 2019 to November 30, 2020) we recruited early midcareer research faculty from U.S. medical schools and teaching hospitals to participate in a randomized controlled experiment. Inclusion criteria were: appointment for 3–14 years at a U.S. medical school or teaching hospital; associate professor or at least two years at rank of assistant professor (or equivalent); and demonstrated competence in securing research funding that included a current or recent first-time NIH R01 or R01-equivalent award, R21 or R34 award, HRSA, ARHQ or other federal agency major grant, K training grant, or recent major foundation or professional organization grant. We excluded those with more than one R01-equivalent award so as to focus on faculty most vulnerable to leaving sponsored research given the high attrition rate from federal funding of first-time R01 awardees [7].

To obtain the sampling frame, NIH RePORTER was searched for all awardees receiving qualifying grants from 2013 to 2019. The sources used did not provide needed information to apply inclusion/exclusion criteria, so recruiting covered all potential subjects located via our search criteria for whom we could find contact information. Hard copy invitations to apply to the Institute were mailed to 4,791 individuals in the recruitment pool, and emails were sent to 5,202 individuals, with most sent both methods of communication (4,438) [22].

Because the study design for the intervention called for 50% participation by persons from underrepresented racial and ethnic groups as defined by NIH [24] (Black/African-American, Hispanic/Latinx, Native American, Alaska Native, and Pacific Islander), additional methods were used for focused recruitment. First, grant titles using words associated with diversity concepts (e.g. “disparities”) were flagged. Second, photos of researchers were located through Internet searches and scored for likely race and ethnicity using Kairos on-line facial recognition software (www.kairos.com) [25]. Third, a list of common Latinx surnames was used to identify possible Latinx researchers. Finally, deans and others with responsibility for diversity at medical institutions were contacted to identify and alert eligible faculty members of the opportunity to participate in the Institute. Those identified as possibly belonging to these underrepresented groups received additional customized emails [22].

Those wishing to participate completed applications that included self-reports of the inclusion criteria for the study and a current CV. Additionally, applicants consented that should they be selected, they would participate in either the initial yearlong intervention group or a delayed intervention group starting the Institute 12 months later. Of 270 applications received, 99 met all inclusion criteria.

**Fig 1:**
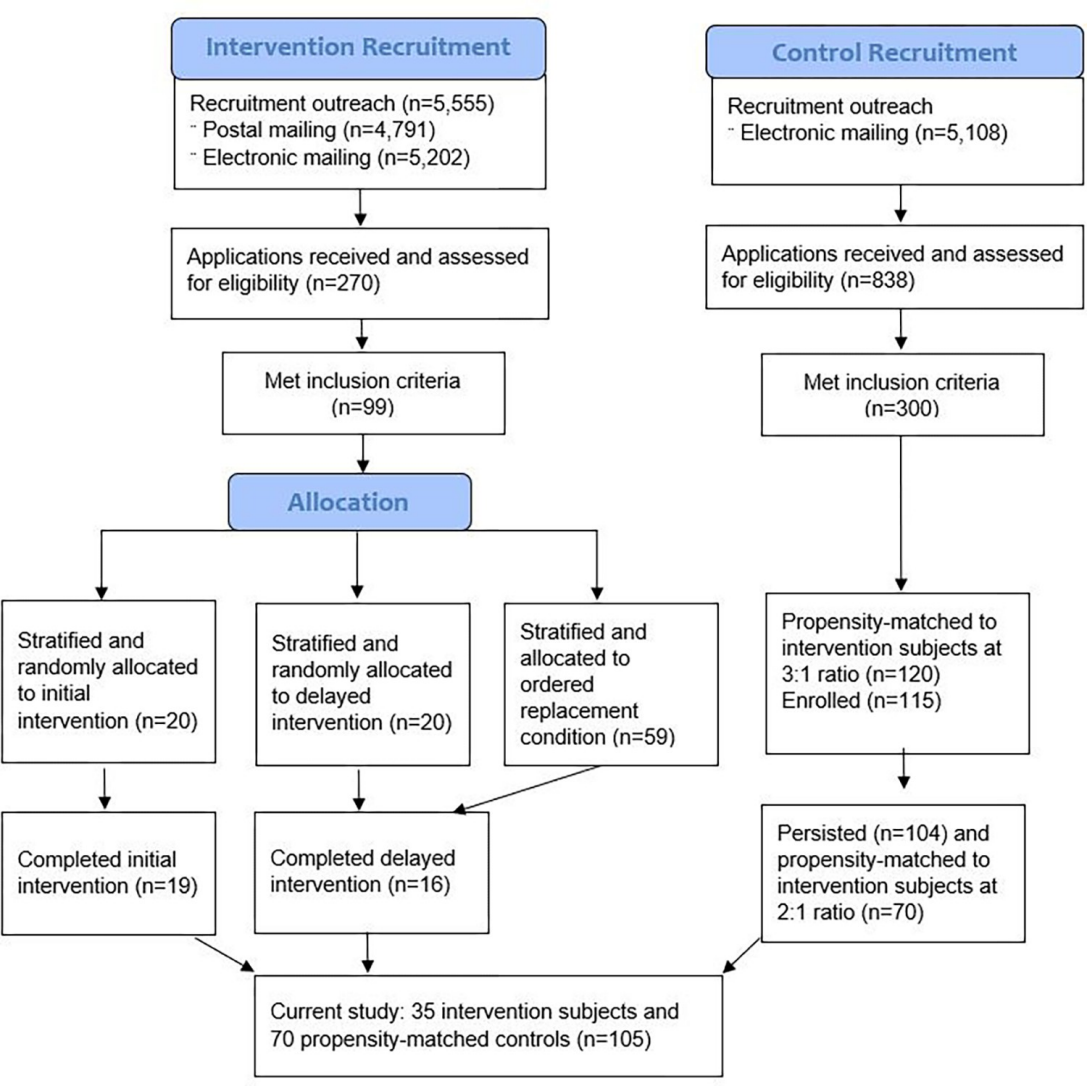
Recruitment and allocation of study subjects. Due to attrition in the delayed intervention group, four subjects were added from the randomized replacement group. 35 subjects completed the intervention and were compared with 70 propensity-matched control subjects at a 2:1 ratio.

Eligible applicants were stratified by three binary characteristics: race and ethnicity (non-underrepresented vs. underrepresented in medicine), gender (male vs. female), and degree (M.D. or M.D.,Ph.D. vs. Ph.D.). The priority was to maintain a 50:50 balance by gender and by race and ethnicity (actual identification of race and ethnicity was obtained from self-report in applications). Within each of the resulting cells, the respondents were put on randomly ordered lists in Excel v.2019 (Microsoft, Redmond, WA) to be assigned to the initial intervention group or the delayed intervention group of 20 subjects each, stratified as described. The remaining members of each list were notified of being placed on a waiting list and used in randomized order to replace any losses, such as failure to accept the offer or schedule modifications due to the SARS-2-CoV pandemic. Fig 1 displays the recruitment and allocation process for the mentoring intervention subjects as well as for the propensity-matched control subjects (described next). For the present analysis, the two intervention groups spanning 2020–2022 were combined.

Because the delayed intervention group would be treated one year after the intervention of the initial group, we would have no control group for long-term follow-up on career markers, such as scholarly productivity and promotions. Furthermore, the randomized design, which was limited by the maximum practical enrollment of the Institute (i.e., 20), was too small for statistical comparisons that were of interest to us. For, example, under the randomized design there would be only five underrepresented female faculty in the intervention group. To address these shortcomings, we conducted a second recruitment—this time for propensity-matched (PM) control subjects—using the database created to recruit intervention subjects. Emails were sent to 5,108 researchers who were invited to participate in the study and offered financial incentives to complete surveys and provide ongoing career information. Of 838 applications received, 300 met inclusion criteria which were relaxed from those used for intervention subjects to allow an optimal matching propensity procedure to determine the best statistical matches; specifically, rank, years of experience, and number of R01 awards were not constrained. Designating a 3:1 match, 120 faculty were statistically matched to the attributes of the 40 intervention subjects using optimal matching through MatchIt [26] in R [27]. Optimal matching, as opposed to nearest neighbor, minimizes total distances between all intervention subjects and their matches [26]. While 120 applicants were matched, five failed to enroll in the study for a total of 115 controls (Fig 1). All intervention participants and propensity-matched control subjects signed a written informed consent form.

In the first year of the study, the Covid-19 pandemic necessitated a delay in the start of the study intervention and a disruption in the schedules and plans of many subjects. Nine intervention subjects across both groups withdrew from the study, partly related to Covid, but only four could be replaced due to the timing of the withdrawals, for an effective sample of 35 intervention subjects. In the control group, 11 subjects withdrew or became inactive, for an effective sample of 104 control subjects.

Given the attrition in both intervention and control groups and, in particular, the addition of replacement subjects to the intervention group, we elected to rematch the control subjects to the intervention subjects, this time designating a 2:1 match (i.e., 70) from within the 104 remaining control subjects. The rematch offered the benefit of not only improving matches for the replacement intervention subjects, but identifying better matches for *all* intervention subjects because we were able to include key measures from the study’s baseline survey as matching variables. For this round of matching, we included the original eight standardized matching variables from application form data: gender, race and ethnicity, degree, rank, years of experience, number of publications, weighted number of grants, and number of R01 or equivalent grants. Additionally, we included eight standardized measures from the baseline survey that reflect key study outcomes: vitality and self-assessed change in vitality; self-efficacy in career advancement, research, and mentoring others; and valuing diversity, cognitive empathy, and anti-sexism and anti-racism skills.

### Intervention

The yearlong facilitated group peer mentoring intervention convened in person quarterly for two- or three-day residential, immersive sessions. Theoretical foundations for the Institute were adult learning theory [28–30], Rogerian psychological principles [31], group theory [32], praxis [33], development of personal awareness [34], cognitive empathy [35], and self-determination theory [36].

**Fig 2:**
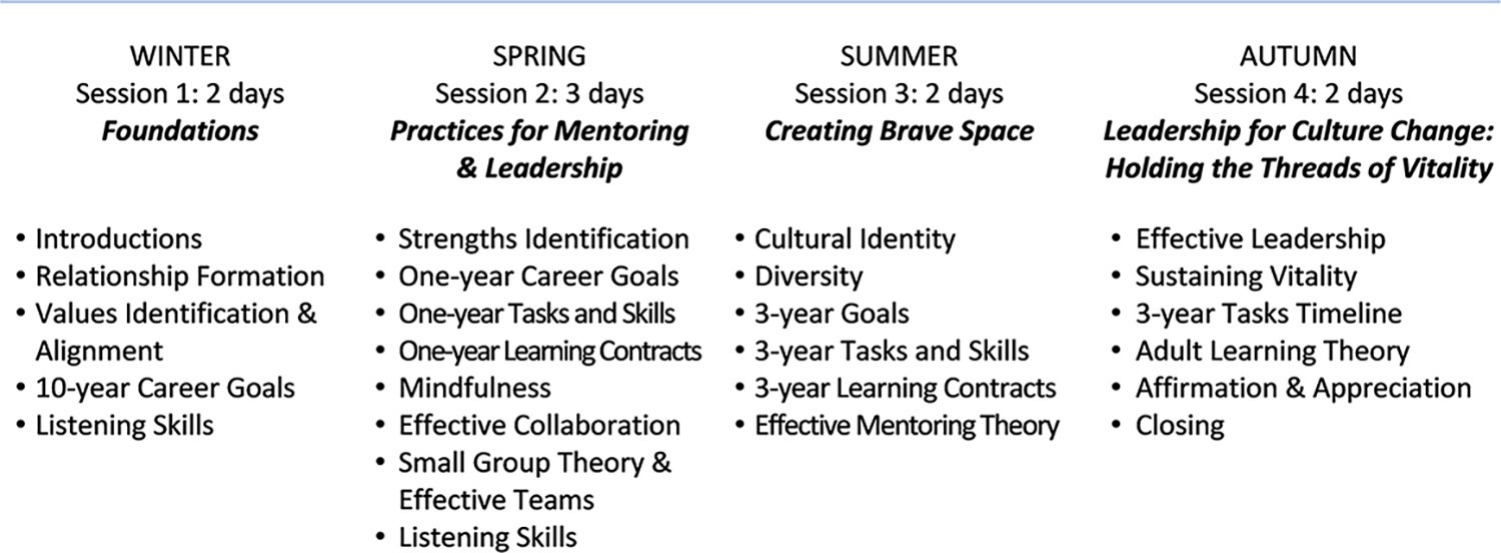
C-Change mentoring and leadership institute curriculum content.

Participants represented multiple disciplines in academic medicine and were from all regions of the U.S. The course was characterized by nonhierarchical peer relationships, empowerment, self-direction, and reflection where each participant simultaneously held the roles of both mentor and protégé. The sessions employed experiential, cognitive, and affective learning methods. The curriculum addressed knowledge and skills essential for leaders and advancement in academic medicine, and for effective mentoring. Curricular content focused on relationship formation, identification of personal core values and their alignment with career and personal goals, listening skills, identification of strengths, mindfulness, effective collaboration and teamwork, appreciation of diversity and cultural self-identity, effective mentoring and leadership models, and sustaining vitality (Fig 2). During each session, participants engaged in a highly structured process of career planning and learning skills in key areas for career advancement [37]. Each participant was guided by their peers through the steps of formulating an explicit written personal career plan that included short- and long-term goals and identification of the tasks and skills to develop in order to attain those goals [37].

Participants mentored each other as peers during frequent dedicated small-group sessions of two or three participants. These assigned small groups of participants changed within each day and between sessions so that everyone had the opportunity to benefit from the perspectives of many peers. Usually, the small groups were followed by debriefing and large group dialog in which participants were invited to speak about their own learning and listen to the described learning of other participants. The facilitators ensured a safe space for dialog within large-group events. The course was writing intensive, and each day, participants wrote about their learning experiences. Relevant key articles were provided for each content area, usually to be read after the session. The facilitators kept to the timed agenda, brought formal attention to the Institute learning culture and stimulated communication within the group.

The initial intervention commenced in December 2020 and the delayed intervention started one year later. Due to Covid-induced travel restrictions, the first two sessions of the initial intervention cohort were conducted by virtual conferencing, and all subsequent sessions employed Covid-19 precautions.

### Outcome measures

Participants in each intervention cohort and their propensity-matched controls were assessed with an on-line survey prior to the Institute (intervention) for baseline measurement or pre-test, and again one year later upon completion of the Institute (post-test). Table 1 shows details of each of the scales used.

**Table 1:** Outcome measures and estimated group means^a^ (unadjusted) pre-intervention and at completion of intervention^b^ for 35 intervention subjects and 70 propensity-matched control subjects.

			Unadjusted mean		Unadjusted mean	
			pre-intervention^c^		post-intervention	
Outcome measures (number of items)	Re-sponse scale	Est^d^ Cron-bach alpha	PM^e^ control group	Inter-vention group	PM^e^ control group	Inter-vention group
			n = 70	n = 35	n = 70	n = 35
**Vitality (4)** *Being energized by work*	1–6	.89	4.96	4.89	4.84	5.07
**Self-assessed change in vitality (4)** *Current vitality compared to one year ago*	1–7	.93	4.28	4.28	4.40	5.24
**Self-efficacy: career advancement (4)** *Self-confidence in ability to advance in career*	1–7	.83	5.33	5.39	5.03	6.01
**Self-efficacy: research (4)** *Self-confidence in ability to be successful in research*	1–6	.87	4.03	3.71	4.04	4.48
**Self-efficacy: mentoring others (7)** *Self-confidence in ability to effectively mentor others*	1–6	.91	4.57	4.27	4.48	4.99
**Valuing diversity:** **a) attitudes (3)** *valuing diversity in work teams*	1–6	.92	5.54	5.66	5.61	5.50
**b) behaviors (4)** *extent of preferential consideration of diversity in recruitment and advancement*	1–6	.87	4.44	4.64	4.24	4.33
**Cognitive empathy (6)** *Ability to identify concerns and discomfort in others*	1–7	.89	5.25	4.95	5.29	5.59
**Anti-sexism and anti-racism skills (4)** *Ability to identify and respond to gender*, *race*, *and ethnicity inequity*	1–7	.84	5.10	5.17	5.10	5.87

^a^ Means calculated using all domain items after imputation.

^b^ Measurement for each intervention group (initial and delayed) and their matched controls was conducted pre-intervention and at completion of intervention.

^c^ Pre-intervention, there were no statistically significant (i.e., no p < .05) differences between intervention and control groups on any of the outcome measures.

^d^ Est = Estimated

^e^ PM = Propensity-matched

#### Primary outcomes: Change in vitality and self-efficacy

To test for group differences on one of our primary outcomes, vitality, we included two scales. One included four items using a six-point ordinal frequency response scale ranging from “never” to “very frequently”–items explored finding work energizing and meaningful, looking forward to coming to work. The scale derived from the validated C-Change Faculty Survey (CFS) [5, 6]. The second vitality scale used the same four items, but asked participants to self-assess their change in vitality over a twelve-month period (i.e., “compared to a year ago”) with responses on a seven-point Likert agreement scale from “very strongly disagree” to “very strongly agree”.

For our second primary outcome, self-efficacy in career advancement, we used two scales. One measured confidence in ability to overcome barriers and progress in career [5, 6], with subjects rating the truth of each statement with an anchored seven-point ordinal scale from “completely false” to “completely true.” A related scale was developed to assess subjects’ perception of their potential for research success. Using an anchored six-point ordinal scale ranging from “not at all confident” to “completely confident,” subjects responded to statements about being successful in research, becoming a leader in research, securing research funding, and maintaining a research network.

#### Secondary outcomes: Cultural awareness and appreciation of diversity

As secondary outcomes, the study sought to understand if Institute participants were more likely than their PM-control counterparts to demonstrate improved cultural awareness and appreciation of diversity. This cross-cultural competence domain was assessed using three scales that measure: 1) cognitive empathy, 2) valuing diversity: a) attitudes and b) behaviors, and 3) anti-sexism and anti-racism skills.

The cognitive empathy scale measured the ability to comprehend others’ experiences. It consisted of a subset of items from a valid long instrument developed by Reniers and colleagues, the QCAE: A Questionnaire of Cognitive and Affective Empathy [22, 35]. Items included characteristics such as ability to predict whether someone is concealing their true emotions, anchored on a seven-point ordinal scale ranging from “completely false” to “completely true” [22].

For our hypothesized outcome of greater appreciation of diversity, we wrote new items to assess various aspects of valuing diversity [22], which resulted in the creation of a measure of valuing diversity focused on: a) attitudes about the benefits of a diverse workforce, and b) behaviors to achieve a diverse workforce (e.g., consideration of diversity in recruitment). Subjects responded to each belief statement on an anchored six-point ordinal scale ranging from “very untrue” to “very true” [22].

A third scale reflected subjects’ assessment of their ability to identify and effectively respond to incidents of sexism and racism (e.g., “I can easily identify gender inequity”). These items used an anchored 7-point ordinal scale ranging from “completely false” to “completely true” [22].

#### Mentoring self-efficacy

We assessed subjects’ confidence in mentoring others using seven items adapted from our published and validated scale: [38] three items addressed professional goals—formulating goals, identifying skills needed as well as specific plans to achieve their goals; two items concerned helping find the resources as well as a sponsor or champion to advance their work; one item was related to helping to define personal goals, and a final item assessed overall confidence in being an effective mentor.

### Data analysis

Table 1 shows the eight study outcome scales and their psychometric properties assessed by item correlation and Cronbach alpha coefficients. To maintain the integrity of the stratified controlled design, all missing data were imputed, although the incidence was very low at <1%. To address missing data at the item level within scales and for completely missing scales, 100 data sets were multiply imputed using chained equations, imputing each item individually before calculating scales. The Stata plug-in ICE [39] was used for computation.

Post-test scores were evaluated via regression. All models included the pre-test or baseline version of the outcome variable (with the exception of Self-assessed change in vitality [40] and the three study design stratifiers as dummy variables: race and ethnicity, gender, and degree. Product-term interactions between intervention (intervention versus control) and each of the stratifiers were used to assess potential differences in intervention effects. All models were estimated using the MI estimate command (for multiply imputed data) in Stata 18 (StataCorp, College Station, TX) [39]. We used the widely accepted standard for effect sizes [41], where an effect size—representing a standardized mean difference between two groups in standard deviation units, or *D*—of 0.3 is considered “moderate” and 0.5 is considered “large”.

Brandeis University Human Subjects Protection IRB approved this study, IRB #19127R-E. (hrpp@brandeis.edu phone: (718) 736 8133.

## Results

This prospective controlled trial included 105 study subjects, with 35 in the intervention group and 70 propensity-matched controls (Fig 1), assessed prior to the intervention for baseline measurement and again at completion. All intervention subjects completed the study’s on-line survey pre- and post-intervention for a 100% response rate at each timepoint. PM controls achieved a 100% response rate at baseline and 97% at intervention completion. At baseline, the intervention and PM control groups were similar on demographics, all eight outcome measures (Table 1), as well as number of publications, research awards, and years in academic medicine (Table 2). The 70 control subjects were compared to the 35 intervention subjects on each of the 16 matching variables to evaluate the success of the optimal matching procedure. Using contingency tables (χ^2^), Mann-Whitney U, or T-test as appropriate, we found no statistically significant difference between control and intervention subjects on any of the 16 matching variables. Therefore, matching appeared successful in creating comparable groups at baseline.

**Table 2:** Characteristics of 35 intervention subjects and 70 propensity-matched control subjects^a^.

Characteristic	PM^b^ control group	Intervention group
	(n = 70)	(n = 35)
	*n (%)*	*n (%)* ^c^
Female	42 (60)	16 (46)
Race and ethnicity: URM^d^	21 (30)	17 (49)
Race and ethnicity by gender		
Non-URM male	21 (30)	9 (26)
Non-URM female	28 (40)	9 (26)
URM male	7 (10)t	10 (29)
URM female	14 (20)	7 (20)
Degree		
Ph.D.	38 (54)	15 (43)
M.D.	26 (37)	15 (43)
Both M.D. & Ph.D.	6 (9)	5 (14)
Rank		
Assistant professor	33 (47)	15 (43)
Associate professor	34 (49)	20 (57)
Professor	3 (4)	0
NIH research award		
K award recipients	39 (56)	16 (46)
R01 award recipients	38 (54)	13 (37)
Both K and R01 recipients	60 (86)	24 (69)
Other major federal research award	27 (39)	18 (51)
Other major award from professional organization or foundation	0 (0)	3 (9)
Mean years in academic medicine in 2019 (SD)	9.1 (4.2)	9.1 (2.6)
Mean number of publications (median, IQR)	31.4 (29.5,19–40)	29.1 (25.0, 14–38)

^a^ There were no statistically significant (i.e., no *P* < .05) differences between intervention and control groups for any of the characteristics by relevant statistical test (χ^2^, Mann-Whitney U, or T-test).

^b^ PM = Propensity-matched

^c^ Some percentages do not add to 100% due to rounding.

^d^ URM = underrepresented in medicine: individuals from racial and ethnic groups that have lower than expected representation in the health‐related sciences as designated by the National Institutes of Health and the National Science Foundation. Notice of NIH’s Interest in Diversity. National Institutes of Health. November 22, 2019. Accessed June 22, 2023 https://grants.nih.gov/grants/guide/notice-files/NOT-OD-20-031.html

At post-test, the intervention group had significantly higher scores (favorable) than the PM control group for seven of the eight outcome measures, controlling for baseline value, gender, race and ethnicity, and degree (Table 3). For all four primary outcomes, the intervention group had significantly higher adjusted post-test scores, with effect sizes as standardized mean differences or *D* of 0.33 for Vitality, 0.67 for Self-assessed change in vitality, 0.70 for Self-efficacy for career advancement, and 0.60 for Self-efficacy for research success. These effect sizes are typically considered moderate to large.

**Table 3:** Estimated differences (regression coefficients) between the intervention group (n = 35) and propensity-matched control group (n = 70) for eight outcome measures at intervention completion (post-test), adjusting for pre-test score and gender, race and ethnicity, and degree^a^.

Outcome measures^b^	Adjusted difference between intervention and PM control groups at post-test (95% CI)		Effect size *(D)*^d^	*P-v*alue
	Difference^c^	95% CI		
***Primary outcomes***				
Vitality	0.28	0.04, 0.53	0.33	0.02
Self-assessed change in vitality^**e**^	0.82	0.29, 1.37	0.67	0.003
Self-efficacy: career advancement	0.90	0.57, 1.24	0.70	<0.001
Self-efficacy: research	0.58	0.28, 0.88	0.60	<0.001
***Secondary outcomes***				
Self-efficacy: mentoring others	0.57	0.30, 0.85	0.81	<0.001
Valuing diversity: a) attitudes b) behaviors	0.19	-0.07, 0.45	0.27	0.15
	0.14	-0.26, 0.55	0.12	0.48
Cognitive empathy	0.53	0.22, 0.84	0.52	0.001
Anti-sexism and anti-racism skills	0.70	0.34, 1.07	0.72	<0.001

^**a**^ Regression analyses conducted with 100 datasets that were imputed using chained equations. Regression models controlled for the pre-intervention (pre-test) value of the outcome variable (excluding Self-assessed change in vitality), gender, race and ethnicity, and degree.

^**b**^ Measurement for each intervention group (initial and delayed) and their matched controls was conducted pre-intervention (pre-test) and at completion of intervention (post-test).

^c^ A positive value is a result favoring the intervention group.

^d^ Effect size *D* is the standardized mean difference calculated from the regression coefficient [41]. A common interpretation of effect sizes is that a value of 0.3 is considered a medium effect and more than 0.5 is considered large.

^e^ The regression model for this outcome excluded the pre-intervention measure [40].

For three of the four secondary outcomes, the intervention group had significantly higher adjusted post-test scores, with effect sizes of *D* = 0.81 for Self-efficacy mentoring others, 0.52 for Cognitive empathy, and 0.72 for Anti-sexism and anti-racism skills. The only nonsignificant results were for Valuing diversity.

These results are consistent with those of our smaller randomized controlled trial, although in the randomized controlled trial the difference in Vitality favoring the intervention group did not reach statistical significance (Effect size *D* = 0.31, *P* 0.20) [22] whereas in this larger non-randomized controlled trial, the difference was similarly moderate but statistically significant (Effect size *D* = 0.33; P 0.02).

### Differences by gender

For all eight outcome measures, the intervention had a nearly identical benefit for men and for women; *P* value for interaction between gender and intervention for all outcomes ranged from *P* 0.10 to 0.99.

### Differences by race and ethnicity

**Fig 3:**
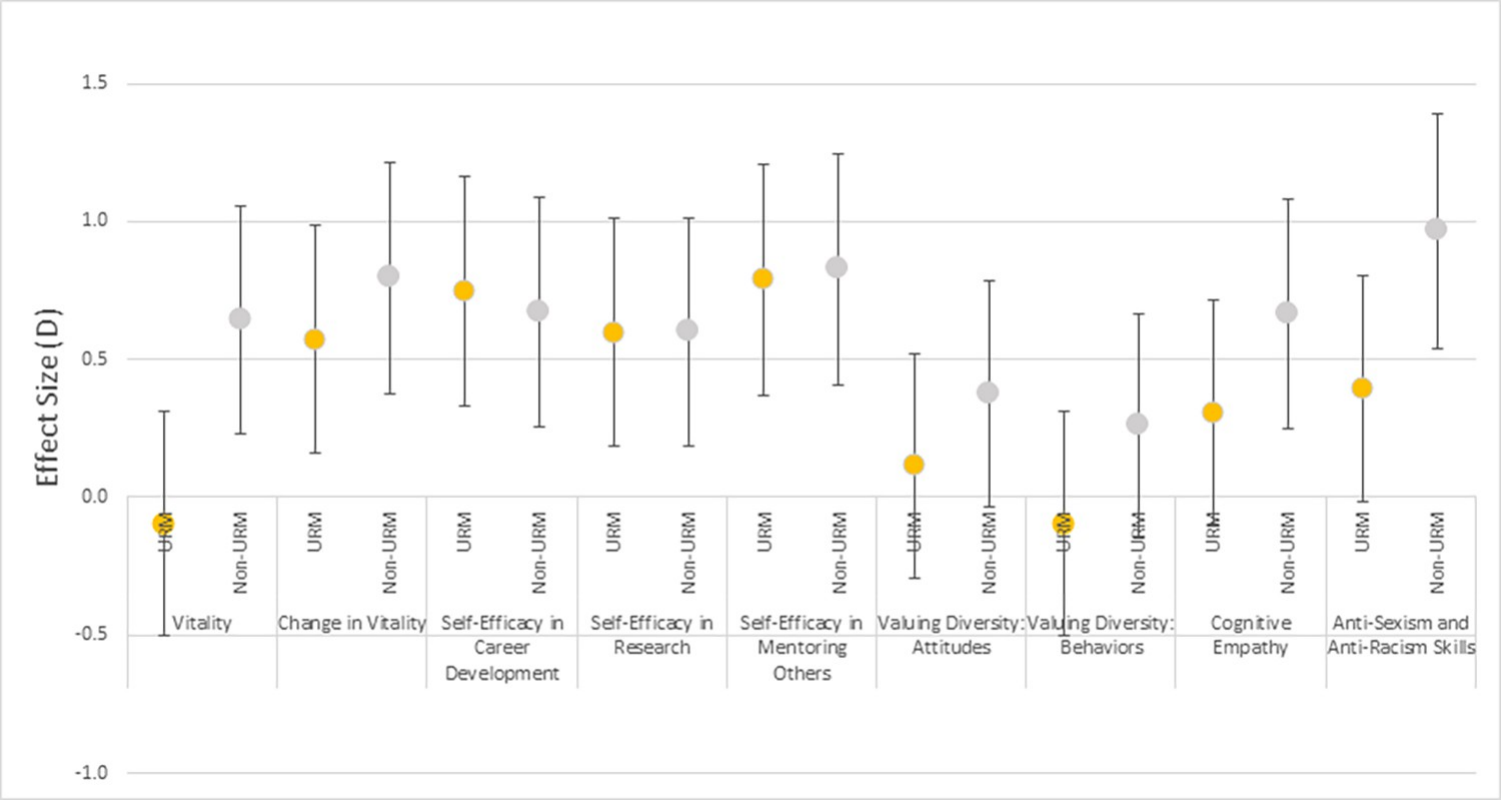
Effect (*D*) of the intervention within demographic subgroups by race and ethnicity for 8 outcome measures, adjusting for pretest values, gender, and degree. ^a^ Regression analyses conducted with 100 datasets that were imputed using chained equations. Regression models controlled for the pre-intervention value of the outcome variable (excluding Self-assessed change in vitality), gender, URM, and degree, and included an interaction term between URM and intervention to assess for potential differences in intervention effects between URM and non-URM faculty subgroups.^b^ Effect size *D* is the standardized mean difference calculated from the regression coefficient [41]. A common interpretation of effect sizes is that a value of 0.3 is considered a medium effect and more than 0.5 is considered large.

For those subjects from underrepresented race and ethnicity groups (URM), the intervention appeared to have no effect on Vitality (Fig 3), whereas for non-URM subjects the intervention significantly improved Vitality (difference in intervention effects between URM and non-URM: 0.6, *P* 0.01). For all other outcomes, including enhanced Self-assessed change in vitality, the intervention effects were not statistically different between URM and non-URM. However, for three secondary outcomes (Valuing diversity, Cognitive empathy, and Anti-sexism and anti-racism skills), the confidence interval for the difference in intervention effect between URM and non-URM includes values that might be considered important (Fig 3), although the *P* value for interaction for all three outcome measures is nonsignificant (range: *P* 0.17 to 0.52).

### Differences by degree

For all eight outcome measures, the intervention had a similar benefit for physician investigators (M.D. degree) and for Ph.D. investigators; *P* value for interaction between degree and intervention for all outcomes ranged from *P* 0.07 to 0.95.

### Sensitivity analysis

We analyzed the data using a random effects hierarchical model to account for clustering among subjects (intervention subject and their two matched controls) and found no difference in results.

## Discussion

The findings of this study comparing outcomes in intervention subjects with PM controls align with and build on our published randomized controlled study of the effects of the Institute [22]. We found that our mentoring intervention worked successfully in enhancing vitality, self-efficacy, and cross-cultural competence broadly across different demographic groups of medical school research faculty. We found similar positive effects on men and women, across race and ethnicity, and for physician and Ph.D. investigators regarding the outcomes of self-assessed change in vitality, self-efficacy, cognitive empathy, and anti-sexism and anti-racism skills of intervention subjects. Our PM control group members were extremely well matched both in terms of their personal and professional attributes, as well as their baseline survey responses, which provides a high degree of confidence in our findings. Valuing diversity was statistically significantly enhanced by the intervention in our randomized controlled study but not in this study. Not surprisingly—we observed high ceiling effects in measurement of valuing diversity at baseline among treated URM faculty, i.e., URM faculty entered the Institute intervention already highly valuing diversity. This may contribute to the less positive demonstrated impact of the intervention in this dimension when comparing intervention and PM control subjects as 50% of our intervention subjects were from URM groups, compared with a smaller proportion of URM faculty that we were able to recruit among PM control subjects. Related to cross-cultural domains, it is notable that cognitive empathy increased significantly across all intervention demographic groups. Cognitive empathy is considered a key requisite for effective cross-cultural engagement. The ability to recognize and address sexism and racism was also increased across all demographic groups after participation in the intervention.

Vitality increased for non-URM faculty controlling for gender and degree. The measured vitality of URM faculty did not increase to statistical significance with the intervention although URM faculty did report increased self-assessed change in vitality. The lack of treatment effect for vitality in URM faculty as compared with their non-URM counterparts, might have several explanations including: a) URM participants might have developed a different understanding of vitality during the intervention; b) it might be harder to change vitality in persons from historically underrepresented racial and ethnic groups; c) the intervention might be less effective in the dimension of vitality; and d) the finding might be due solely to sampling error.

A limitation is that randomization was not used to generate the control group. However, our propensity -matched control group was similar to the intervention group on basement measurement of all outcomes indicating that matching was successful. Another potential limitation is that the sample might not be representative of the target population of early midcareer medical research faculty because the study relied on volunteers who sought enrollment in the Institute (intervention group) or who agreed to participate (control group) with compensation for completing surveys. It is impossible to know either the direction or strength of volunteer bias on any of the variables studied: those seeking to attend the Institute might on average be high achievers looking to further advance their careers, or they might be struggling with their career choices and hoping for a boost. The Institute used two highly trained, experienced facilitators. It is uncertain how much of the positive intervention effect is attributable to the specific characteristics of the facilitators. We have not established the sustainability of the intervention effects. Further research is underway to replicate these results and assess whether the positive effects are sustained.

## Conclusion

A notable aspect of the Institute is its blending of traditional career advancement indicators such as vitality and self-efficacy, with the goal of attaining cross-cultural competence. The group nature of the intervention is a structural attribute designed to reliably provide for varying and unpredictable needs of different individuals. Whereas some faculty may need nurturing in terms of their self-efficacy, others may need education in terms of successful promotion, cross-cultural engagement, or mentoring proficiency. We first established the benefit of the Institute in a small, randomized trial (n = 40 subjects) and then replicated the results in this larger (n = 105 subjects) propensity-matched controlled trial, while using the larger study size to verify that the Institute’s multiple benefits apply to researchers from a range of demographic subgroups. Although there was some overlap in the subjects included in our randomized trial and our larger propensity-matched trial, that overlap was 54% (19 of 35) for those in the current study’s intervention group and 0% for those in the control group. Our findings provide robust and reliable evidence of the efficacy of this group peer mentoring model to engage and sustain early midcareer research faculty from diverse backgrounds.
